# The emerging roles of N6-methyladenosine (m6A) deregulation in polycystic ovary syndrome

**DOI:** 10.1186/s13048-025-01690-7

**Published:** 2025-05-23

**Authors:** Leyi Jiang, Jiaying Xiao, Liangzhen Xie, Feifei Zheng, Fangliang Ge, Xue Zhao, Ruonan Qiang, Jie Fang, Zhinan Liu, Zihan Xu, Ran Chen, Dayong Wang, Yanfeng Liu, Qing Xia

**Affiliations:** 1 Department of Gynecology, Dongzhimen Hospital, Beijing University of Chinese Medicine, Beijing, China; 2 Department of Neurosurgery, the Second Affiliated Hospital of Zhejiang University School of Medicine, Hangzhou, Zhejiang 310000 China; 3 Department of Neurosurgery, Ningbo Hospital, Zhejiang University School of Medicine, Ningbo, 315010 China; 4 Department of Biochemistry and Molecular Biology, School of Basic Medical Sciences, Harbin Medical University, Harbin, Heilongjiang, China; 5 Department of Gynecology, Heilongjiang University of Chinese Medicine, Harbin, Heilongjiang, China

**Keywords:** Polycystic ovary syndrome, N6-methyladenosine, Reproductive disorders, Chronic low-grade inflammation

## Abstract

Polycystic ovary syndrome (PCOS) is an endocrine metabolic syndrome characterized by ovulation disorders, hyperandrogenemia, and polycystic ovaries, which seriously affect the psychological and physical health of childbearing women. N6-methyladenosine (m6A), as the most common mRNA epigenetic modification in eukaryotes, is vital for developing the female reproductive system and reproductive diseases. In recent years, an increasing number of studies have revealed the mechanisms by which m6A modifications and their related proteins are promoting the development of PCOS, including writers, erasers and readers. In this work, we reviewed the research progress of m6A in the pathophysiological development of PCOS from the starting point of PCOS clinical features, included the recent studies or those with significant findings related to m6A and PCOS, summarized the current commonly used therapeutic methods in PCOS and the possible targeted therapies against the m6A mechanism, and looked forward to future research directions of m6A in PCOS. With the gradual revelation of the m6A mechanism, m6A and its related proteins are expected to become a great field for PCOS treatment.

## Introduction

**Fig. 1:**
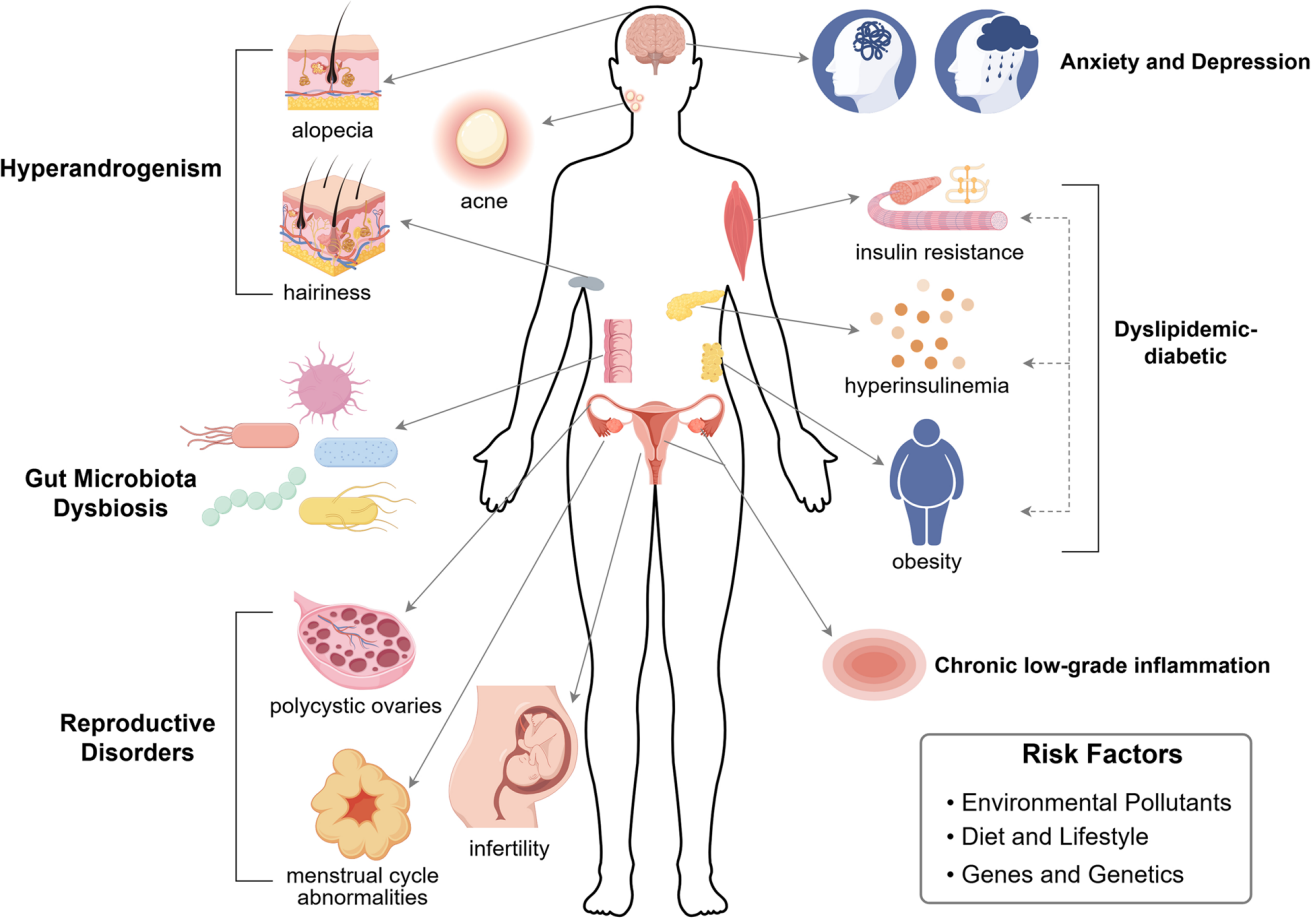
Three broad categories of symptoms and risk factors for PCOS

Polycystic ovary syndrome (PCOS) is a disease of reproduction, metabolism, and mental health, which affects 6%–13% of women of reproductive age worldwide [1–4]. Besides these three characteristics in the Rotterdam criteria, patients with PCOS experience obesity, insulin resistance, type 2 diabetes, and other disorders of glucolipid metabolism [5]. Some studies show that PCOS can cause or aggravate anxiety and depression in patients [6, 7]. Pregnant women with PCOS not only have a greater incidence of gestational diabetes and hypertension, but their offsprings are also more likely to develop reproductive, metabolic and psychological abnormalities [8, 9]. Briefly, PCOS has seriously impinged on women's and human long-term health (Fig. 1). Consequently, it’s extremely urgent to unravel the new pathogenesis and discover new therapeutic targets for PCOS to improve treatment options for women and their offsprings.

The relationship between m6A and PCOS was addressed a few years ago, and as research progressed, m6A was found to play a key role in the pathologic process of PCOS. N6-methyladenosine (m6A) modifications as a part of epigenetics, its mapping, and biological characterization have been extensively studied in recent years. m6A is involved in all critical steps of mRNA processing, translation, and degradation at the molecular biological level. And it has been reported to affect normal hematopoiesis, neurological and embryonic development, immune response, psychiatric disorders, metabolism, and cardiovascular diseases, among many others at the physiological level [10]. Epigenetics modifies gene expression without altering gene sequences to alter biological traits, including DNA methylation, non-coding RNA regulation, chromosome rearrangement, and histone modification. Among them, the RNA methylation is particularly essential [11–13]. There are various RNA modifications, of which more than 160 have been identified [14, 15]. In higher eukaryotic cells, m6A is the most enriching, prevalent, and conservative internal co-transcriptional modification, with about three modified adenosine residues in each transcript [16].

In patients with PCOS, ovulation is suppressed, and endocrine and reproductive functions are also affected. In addition, the risk of reproductive system tumors and psychological abnormalities is increased. As PCOS has already seriously endangered women's long-term physical and psychological health, and the situation will become more serious in the future. However, no cure for PCOS has yet been found, with more lifestyle management and symptom-specific comprehensive treatments to be found [17–21]. Although some advances were made in the study of m6A in female reproductive disorders, it still has much potential for exploration in PCOS, while few reviews have addressed m6A in relation to PCOS. Hence, we summarize and focus on the latest research progress on m6A modifications and their role in pathogenesis, listing some conventional treatment modalities for PCOS and targeted therapies for specific pathogenetic mechanisms. Moreover, the article looks at emerging directions for future m6A researches in PCOS. By reviewing and summarizing previous studies, we are able to gain new perspectives on how to overcome PCOS.

### m6A-related proteins

The m6A-associated proteins include writers, erasers, and readers. Writers, the methyltransferase complex, include the methyltransferase-like METTL3, METTL14, Vir-like m6A methyltransferase-associated (VIRMA/KIAA1429), Wilms-tumor associating protein (WTAP), RNA-binding motif protein 15 (RBM15 A/B), zinc finger CCCH domain-containing protein 13 (ZC3H13), and Cbl proto-oncogene E3 ubiquitin protein ligase-like 1 (CBLL1/HAKAI). Erasers act as demethylases responsible for removing m6A, like Obesity-associated protein (FTO) and alkB homolog 5 (ALKBH5). Contrarily, readers, recognize m6A-modified RNAs, including YTH domain family proteins (YTHDF1/2/3), YTH domain-containing proteins 1–2 (YTHDC1/2), heterogeneous nuclear ribonucleo-protein A2B1 (HNRNPA2B1) [22], insulin-like growth factor 2 mRNA binding proteins (IGFBP1/2/3), eukaryotic initiation factor 3 (eIF3) and Leucine Rich Pentatricopeptide Repeat Containing (LRPPRC) [10, 23–26].

### The m6A role in PCOS pathologic development

#### m6A-related proteins and reproductive disorders in PCOS

PCOS is the most common cause of anovulatory infertility, and our review of the literature revealed that the two main reproductive problems are ovulation and endometrium (Table 1).

**Table 1:** The role of m6A in reproductive system of PCOS

m6A types	Regulators	↑/↓regulated in PCOS	Functions in Reproductive System of PCOS	Ref
Writers	METTL3		Expressed during follicle development and is required for female fertility	[27]
	METTL3		Maintaining maternal mRNA stability in oocytes, and is required for murine oocyte maturation and maternal-to-zygotic transition	[28]
	METTL3		Participating in oocyte maturation by regulating m6A modification of Itsn2	[29]
	METTL3	↑	In PCOS– Promoting the inflammation of the ovaries/ ovulatory dysfunction	[30]
	METTL14		Playing a crucial role in successful implantation by precisely regulating both ERα signaling and innate immunity in the uterus	[31]
	WTAP		Required for maintaining normal levels of inflammation in the body	[32]
	WTAP	↑	In PCOS–Stabilizing the mRNA of inflammasome component ASC in GC methylates, activating Caspase-1, inducing GC pyroptosis	[32]
	KIAA1429		Required for folliculogenesis and its deletion in oocytes leads to abnormal apoptosis and proliferation of granulosa cells	[33]
	KIAA1429		Colocalizing with SRSF3 in nuclear speckles and influences the localization of SRSF3 and YTHDC1	[33]
	KIAA1429	↓	In PCOS–Regulating the alternative splicing of oogenesis-related transcripts	[33]
Erasers	FTO		Strongly related with obesity by its impact on feeding behavior and energy expenditure	[34]
	FTO	↑	In PCOS—Linked to ovarian GC dysfunction through flotillin-2 upregulation	[35]
Readers	YTHDC1		Required for embryo viability	[36]
	YTHDC1		Inactivation of which is embryonic lethal and causes oocyte maturation arrest and female sterility	[36]
	HNRNPs		Dysregulated in endometriosis patients and elevated in placental trophoblasts of preeclampsia patients	[17]
	IGFBP1/2/2		Regulating the maternal mRNA abundance during oogenesis	[27]
	IGFBP1/2/3	↓	In PCOS–The levels of which is involved in the selection of the dominant follicle	[37]

#### Ovulation process and m6A-related proteins

Oocyte development consists of three stages: growth, maturation and ovulation. Each follicle's fate is determined by endocrine and paracrine factors. During adolescence, only a few follicles go into preovulation due to cyclic stimulation by gonadotropins. Most of them fail to mature and are eliminated, and only one develops into a dominant follicle. m6A modifications are essential for oocyte and syncytial maturation. Maternal mRNA is transcribed and accumulated in the early oocyte. The accumulation of mRNA is critical and is used for protein synthesis in the oocyte during the oocyte-to-embryo transition, and even in the embryo to maintain post-fertilization development. After the luteinizing hormone (LH) peak, the degradation of maternal mRNA begins, and most polyadenylated mRNA is lost before ovulation. It is thus clear that the transition of mRNA from stable to unstable plays an important role in oocyte cytoplasmic maturation and embryonic transformation, where m6A modifications are essential for oocyte and syncytial maturation. It has been shown that maternal mRNA translation efficiency is reduced in METTL3-inactivated oocytes [28]. Furthermore, several researchers have found that GV oocyte arrest is associated with METTL3 deletion, which impairs GV oocyte development but does not affect the transformation of primordial follicles to actively growing follicles. Subsequently, the METTL3/IGF3BP3-m6A-Itsn2 signaling axis was found to be involved in egg development [27]. METTL3 participate in oocyte maturation by regulating m6A modification of Itsn2 [29], and the levels of IGFBPs are involved in the selection of the dominant follicle [37]. In addition, due to the large number of alternative splicing defects in YTHDC1-deficient oocytes, the primordial stage oocytes are surrounded by only one granulosa layer, resulting in oocytes from Ythdc1fl/-Ddx4-Cre ovaries blocked in the primordial stage [36]. Moreover, oocytes lacking KIAA1429 lose the ability to resume meiosis, leading to follicular dysplasia and even infertility in women. KIAA1429 deletion leads to reduced localization of YTHDC1 and SRSF3 (serine/arginine-rich splicing factor), but SRSF3-associated and YTHDC1-associated conformations were observed in exons adjacent to the cleavage site. Additionally, KIAA1429 participates in posttranscriptional regulation by mostly regulating (alternative splicing) AS in oocytes [33]. Thus, its deletion may lead to abnormal RNA metabolism by interfering with exons. What’s more, researchers have found that environmental impact factors, like constant exposure to light, decrease the oocyte maturation rate by decreasing m6A fluorescence [38].

In PCOS, competing follicles stop growing prematurely and degenerate [39]. The researchers also found that several pathways associated with follicular development were enriched in PCOS, such as the p53 signaling pathway, the FOXO signaling pathway, the hippo signaling pathway and the PI3K-Akt signaling pathway [40].

#### Endometrial receptivity and m6A-related proteins

Women with PCOS are usually infertile, especially those with ovarian failure and recurrent miscarriages, and restoration of ovarian function can’t improve their reproductive potential [41]. Studies have found that the endometrium of women with PCOS is very different from that of women without PCOS, and these alterations include aberrant expression of steroid hormone receptors [42], aberrant regulation of enzymes and metabolic pathways that disrupt the endometrial environment [43], and a different protein composition. The main factors in the human endometrium known to be good markers of endometrial tolerance are IGFBP-1, EGF, TGFβ1, LIF, MMP 9, and integrin β3. A recent study suggests that high TT may alter endometrial tolerance by decreasing the expression of IGFBP-1 and LIF-1. This group of patients has a thinner endometrium, a lower pregnancy rate and a higher miscarriage rate [44]. In addition, placental abnormalities are strongly associated with early embryonic death and developmental abnormalities. In the placental trophoblast of patients with spontaneous abortion, the researchers observed significantly reduced levels of the demethylase FTO and the leukocyte antigen HLA-G, as well as a reduction in the target mRNA for YTHDF2. This suggests that FTO-induced aberrant methylation in chorionic cells alters immune tolerance and angiogenesis at the maternal–fetal interface, ultimately leading to miscarriage [45]. Similarly, the overall m6A modification level of mRNAs was significantly reduced in chorionic cells from patients with recurrent spontaneous abortion. Knockdown of ALKBH5 was also found to reduce CYR 61 mRNA stability, hence inhibiting trophoblast cell proliferation and invasion at the maternal–fetal interface in early pregnancy [46]. Therefore, it is reasonable to hypothesize that infertility in PCOS patients with endometrial abnormalities is likely to be caused by the above m6A-related proteins.

#### m6A-related proteins and chronic low-grade inflammation in PCOS

Chronic inflammation is present in the body of patients with PCOS, which can lead to ovulation disorders and disorders of glucolipid metabolism. elevated inflammation in patients with PCOS alters Anti-Müllerian hormone (AMH) levels, leading to disturbances in glucose and lipid metabolism [47]. This condition increases the risk of insulin-receptor (IR) and androgen synthesis, disrupting regular ovulation and fertilization progress in women and causing menstrual disturbances and irregular ovulation [48]. It has been experimentally demonstrated that higher cytokine and chemokine concentrations in chronic inflammation of the ovaries lead to follicular development disorders [4, 49]. Researchers found that Interleukin (IL)-17A, IL-23, and IL-33 were significantly increased in patients with PCOS, and IL molecules may be involved in the pathogenesis of PCOS in concert with ADAMTS [50]. In addition, tumor necrosis factor-α (TNF-α) may also be an indicator of chronic inflammation in PCOS. Studies have found that overweight patients with PCOS have higher levels of tumor necrosis factor-α (TNF-α) than controls, and obesity would exacerbate this inflammation. On the one hand, abnormal expression of inflammatory factors in the peripheral circulation and in the ovaries of patients with PCOS may lead to immune and ovulatory dysfunction [51, 52]. At the same time, immune system dysfunction may further affect follicular development and ovulation [53]. Additionally, studies have demonstrated that polymorphisms in the VDR gene increase androgen levels in the pathogenesis of PCOS by regulating the production of inflammatory cytokines.

**Fig. 2:**
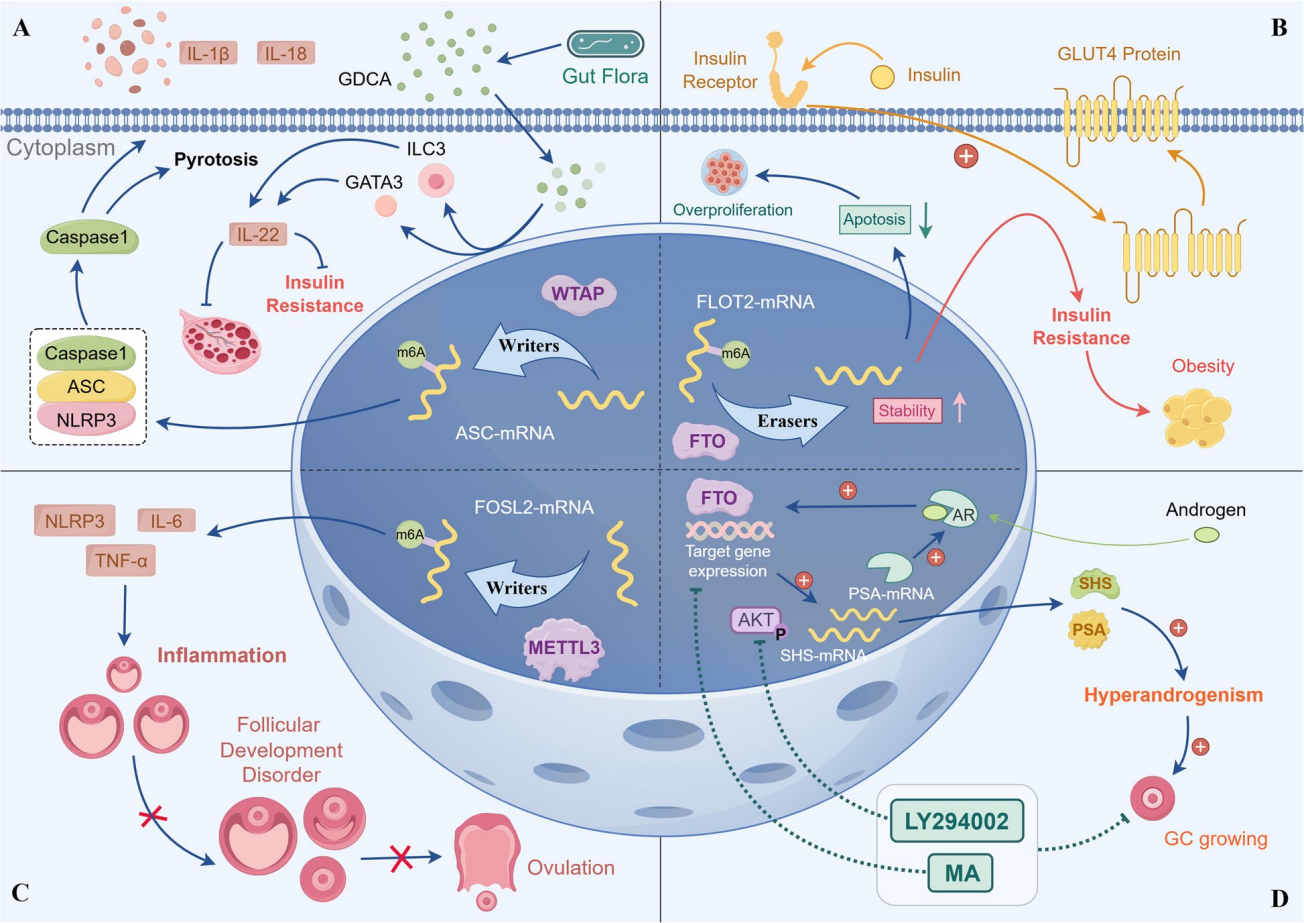
The emerging roles of N6-methyladenosine (m6A) deregulation in polycystic ovary syndrome. **A** WTAP exacerbates GC pyroptosis through hyperactivation of inflammatory vesicles: WTAP acts as a mediator for N6-methylating the NLRP3 inflammasome component ASC, thus increasing the ASC RNA stability, leading to PCOS over-activation in GC; Plumbagin rescues by inhibiting WTAP. Glycodeoxycholic acid induced intestinal group 3 innate lymphoid cell IL-22 secretion through GATA binding protein 3, and IL-22 in turn improved the PCOS phenotype. **B** FTO promotes GC hyperproliferation and leads to insulin resistance and thus obesity by inhibiting GLUT4 transport: FTO overexpression can promote cell proliferation, inhibit cell apoptosis, and induce insulin resistance in GC. FTO reduced the m6A level of FLOT2 mRNA and increased its mRNA stability of FLOT2. In contrast, FLOT2 loss reduced the effect of FTO overexpression on the proliferation, apoptosis, and insulin resistance of GC. Insulin can induce GLUT4 transfer to the plasma membrane, and FTO over-expression inhibits GLUT4 transport induced by insulin in KGN cells, resulting in insulin resistance. **C** METTL3 mediates KGN cells inflammation leading to ovulation disorders: METTL3 overexpression promotes methylation of FOSL2, which causes an increase in NLRP3, IL-6, and TNF-α, ultimately leading to a chronic inflammatory state in the ovary; Butyrate modulates METTL3 to ameliorate. **D** FTO causes hyperandrogenemia through the AKT pathway and exacerbates it via positive feedback: AR activation induces FTO upregulation, and the interaction of upregulated FTO, in turn, with AR, promotes the expression of the AR signaling pathway and steroid synthase, creating a positive feedback mechanism that promotes hyperandrogenemia in PCOS. FTO inhibitors were also found to prevent androgen excess and cell hyperproliferation by blocking the AR-AKT signaling axis in GC; Combination of MA with AKT inhibitor LY294002 synergistically inhibited GCs cell survival through the induce of cell cycle arrest. Abbr: WTAP, Wilms-tumor associating protein; FLOT2, flotillin-2; ASC, caspase-recruitment domain; NLRP3: NOD-like receptors containing pyrin domains; FTO, obesity-associated protein; GLUT4, glucose transporter type 4; METTL3, methyltransferase-like 3; FOSL2, a member of the AP1 family; AR, androgen receptor; PSA, prostate-specific antigen; SHS, steroid hormone synthetase; MA, FTO-specific inhibitor; LY294002, AKT inhibitor; GC, granulosa cell

The m6A modification can regulate cellular inflammation by regulating genes associated with inflammation [54]. RNA methyltransferase METTL3, for example, regulates the nuclear factor kappa-light-chain-enhancer of activated B cell inflammation by raising the Tumor necrosis factor receptor-associated factor (TRAF6) modification level of m6A [55]. Recently, Liu et al. found that METTL3 induced granulosa cell inflammation by mediating m6A modification of FOSL2 (a member of the AP1 family), in which the overexpression of METTL3 promotes the methylation of FOSL2, leading to an increase in NLRP3, IL-6, TNF-α, and ultimately a chronic inflammatory state in the ovary. This can be improved by butyric acid treatment [30] (Fig. 2C). A recent study showed that METTL3 also mediates granulocyte inflammation by upregulating CD36 levels [56]. The end point of chronic inflammation is fibrosis. In contrast, silencing of METTL3 inhibited ovarian fibrosis by downregulating GPX4 [57]. Additionally, it has been shown that the higher inflammatory state in patients with PCOS is likely caused by cellular pyroptosis hyperactivation [32], and WTAP is involved in cellular pyroptosis and inflammation by regulating NLRP3 m6A [58]. Cellular pyroptosis is a new pro-inflammatory mode of cell death that is distinct from apoptosis in terms of morphological changes and cell death mechanisms, and it has been reported to be associated with diseases such as Alzheimer's disease and type 2 diabetes mellitus (T2DM) [59]. It is characterized by its dependence on caspase-1 and is accompanied by the release of large amounts of pro-inflammatory factors. To activate inflammatory vesicles, it is necessary to assemble the pyrin domain of NLRP3 and the pyrin domain of the apoptosis-associated speck-like protein containing a caspase-recruitment domain (ASC) [60]. The assembly of the NLRP3 complex leads to cleavage and activation of pro-Caspase 1 and the release of IL 1β and IL-18, which in turn recruits more inflammatory cells and amplifies the inflammatory response [61–63]. Mechanistic studies have shown that granulosa cells (GC) inflammasome hyperactivation is caused by the upregulation of WTAP, a key regulator of the RNA N6-methylase complex. WTAP acts as a mediator for the N6 methylation of the NLRP3 inflammatory component ASC, which in turn increases the stability of ASC RNAs and leads to the hyperactivation of inflammatory vesicles in the GCs of PCOS [32] (Fig. 2A).

In addition, dysregulation of RNA expression in peripheral blood mononuclear cells (PBMCs) may contribute to an increased risk of PCOS and act as a potential diagnostic biomarker [64]. Meanwhile, studies on competitive endogenous RNA networks (ceRNETs), including long non-coding RNAs (lncRNAs), microRNAs (miRNAs) and circular RNAs (circRNAs), are also promises to provide new biomarkers and effective therapeutic targets for PCOS [65].

#### m6A-related proteins and obesity and insulin resistance in PCOS

Obesity enhances the probability of developing insulin resistance, glucose intolerance, compensated hyperinsulinemia, and dyslipidemia and increases serious pregnancy complications [66]. Overweight women with PCOS have more severe symptoms, with more serious menstrual irregularities, infertility, abortion, gestational diabetes, pregnancy-induced hypertension, prematurity, clinical and biomedical hyperandrogenism, glucose intolerance or T2DM, and metabolic syndrome [67–69]. A US study followed changes in glucose tolerance in 71 US women with PCOS and 23 controls for a mean time of three years. All subjects were normoglycemic at baseline. The study found that women with PCOS had a 16 per cent per person-year conversion rate from normoglycemia to impaired glucose tolerance (IGT) and a 2 per cent per person-year conversion rate from baseline IGT to T2D. In comparison, only five women in the control group converted from normoglycemia to IGT [70]. It has also been suggested that patients with PCOS may suffer from diminished insulin lipolysis, which in turn leads to elevated levels of TNF-α and high-sensitivity C-reactive protein (CRP), ultimately resulting to β-cell dysfunction and insulin resistance [71, 72].

The fat mass and obesity-related gene (FTO) is the first gene found to be strongly associated with adiposity. FTO is a 2-oxoglutarate-dependent nucleic acid demethylase, which is the ninth member of the ALKB protein family (also known as ALKBH9). Owing to the strong affinity for 3-methylthymine (3-meT) and 3-methyluracil (3-meU) single-stranded DNA and RNA [34], it can demethylate m6A and N6, 2'-O-methyladenosine (m6Am) in mRNA, m6A in U6RNA, m6Am in snRNAs, and N1-methyladenosine (m1A) in tRNA [73]. FTO is closely related to PCOS. Firstly, FTO is overexpressed in patients with PCOS, and its expression is highly correlated with energy homeostasis. Studies have also shown that the effect of FTO on BMI in women with PCOS is more than twice the effect found in large population studies [74]. To confirm the role of FTO in regulating energy homeostasis, the researchers first detected that FTO mRNA was highly expressed in key regions controlling energy homeostasis in the mouse brain, such as the arcuate nucleus (AN), paraventricular nucleus, dorsal medial nucleus, and ventral medial nucleus. Then, using ad libitum feeding, fasting and fasting and intraperitoneal injection of leptin as variables and grouping, they found that the expression of FTO mRNA in the AN of fasted mice was reduced by about 60% and was irreversible (not recoverable after leptin supplementation). The results suggest that FTO is strongly associated with obesity by influencing feeding behavior and energy expenditure [35]. Further studies found that FTO may regulate energy homeostasis and food intake by acting through NPY1R and BDNF (associated with regulation of food intake and energy homeostasis), which is interacting with CaMKII (a member of the serine/threonine protein kinase family) to participate in the cAMP response element-binding protein signaling pathway and ultimately inducing NPY [75]. Secondly, FTO overexpression can promote cell proliferation, inhibit cell apoptosis and induce insulin resistance in GCs. Earlier research has revealed how FTO induces insulin resistance. Under normal circumstances, insulin can induce glucose transporter type 4 (GLUT4) transfer to the plasma membrane, and FTO over-expression inhibits GLUT4 transport induced by insulin in KGN cells, resulting in insulin resistance. This was achieved by FTO decreasing the m6A level of flotillin-2 (FLOT2) mRNA and increasing its mRNA stability (Fig. 2B). In contrast, FLOT2 deletion reduced the effect of FTO overexpression on the proliferation, apoptosis, and insulin resistance of GCs. FLOT2 and FLOT1 belong to the family of flotillin/reggie proteins and are closely correlated and co-expressed. FLOT 1 was reported to be involved in the regulation of various cellular activities such as protein transport, insulin signaling and cell proliferation. Therefore, it is reasonable to hypothesize that FLOT 2 is also involved in the development of PCOS by influencing these cellular processes [76]. Thirdly, FTO is probably involved in the molecular mechanisms of T2DM in PCOS patients. Each unit increase in FTO mRNA levels was associated with a 2.797-fold increase in the risk of developing T2DM [77]. Researchers also found that FTO can facilitate mRNA expression of FOXO1, fatty acid synthase (FASN), G6Pase catalytic subunit (G6PC), and diacylglycerol acyltransferase 2 (DGAT2), whose increased expression is closely associated with hyperglycaemia and dyslipidaemia in T2DM patients [78]. Lastly, polymorphisms in FTO are likely to be one of the reasons why obesity characteristics of PCOS patients vary by country and ethnicity, which can affect adipocyte differentiation and function by changing the activity of neighboring genes, including iroquois homeobox-3 (IRX3) and IRX5. For example, rs1421085 can disrupt the binding of the ARID5B deterrent protein, which causes the depression of IRX3 and IRX5, leading to the browning of white fat cells, increased fat storage, and weight gain [79, 80]. In addition, different FTO SNPs can also lead to fat accumulation in different body parts in PCOS patients. For instance, FTO variants were associated with obesity, but there was no association between insulin resistance and glucose intolerance among young Korean women [81]. However, in European and Polish women with PCOS, rs9939609 was nevertheless associated with elevated BMI and impaired glucose tolerance [82, 83]. FTO polymorphisms may be a common link between PCOS, T2DM and obesity. Therefore, can we use certain drugs to alter the microenvironment in which genes are located and change the phenotype of certain genes?

#### m6A-related proteins and hyperandrogenism in PCOS

Hyperandrogenism is vital for the pathophysiological mechanisms of PCOS, and there is a causal relationship between the increased biological activity of testosterone and PCOS, T2DM, and endometrial carcinoma [84]. The major cause of PCOS is hyperandrogenism, which is the most inherited phenotype of PCOS [85, 86]. This has long been reported and well-documented. A genetic study of patients with PCOS and their family sisters found that 115 sisters of 80 patients with PCOS all had hyperandrogenemia, with regular or irregular menstruation, but all had LH abnormalities [87]. In addition, hyperandrogenemic women with PCOS also show abnormal endometrial development. High levels of androstenediol have a pro-proliferative effect while high testosterone levels have an antiproliferative effect [88]. Besides, high levels or strong activity of androgen can affect the formation and function of the endometrial pinopodes, which are hormone-dependent protrusions of the apical plasma membrane of the endometrium interrelated to endometrial receptivity. A rat-based study found that high levels of testosterone resulted in reduced uterine pinopodes and L-selectin ligand (MECA-79) expression at the time of uterine capacitation, leading to failure of blastocyst implantation [89]. Furthermore, endometrial Wilms tumor suppressor gene 1 (WT1) is down-regulated in the secretory phase endometrium of ovulatory hyperandrogenic PCOS women and associated with androgen levels and expression of several proteins(e.g., epidermal growth factor receptor Bcl-2 and p27) involved in apoptosis [90]. WT1 can also regulate AR expression, and the success of metaplasia and endometrial tolerance in PCOS patients may be jeopardized by an altered balance between WT1 and AR in the endometrium [91]. Apart from that, several experiments have shown that hyperandrogenemia impairs endometrial function, either directly or due to hyperinsulinemia. For specific endometrial pathways, hyperandrogenism only plays a role in the presence of hyperinsulinemia, such as the impaired endometrial lipocalin signaling pathway [92], and DHT-enhanced up-regulation of prokineticin 1 (PROK1) in human embryonic stromal cells (hESC) only in combination with insulin [93]. Besides, testosterone significantly inhibited IRS-1 mRNA, IRS-1, and GLUT-4 protein expression in endometrial glandular epithelial cells [94]. Interestingly, Li et al. found that the mRNA levels of IR, IR substrate (IRS) 1, and IRS2 were significantly elevated in the endometrium of patients with PCOS (PCOSE). In addition, they found that chronic exposure to insulin or DHT abnormally increased IRS1/IRS2 phosphorylation as well as GLUT1 and GLUT12 protein levels in hESC, suggesting that hyperandrogenism is also involved in influencing insulin signaling and glucose metabolism. Moreover, the expression of GLUT1 and GLUT12, and the decidualization markers IGFBP1 and prolactin, was suppressed by DHT during decidualization in vitro [95].

Elisabeth et al. provided the first evidence that FTO variants are associated with hyperandrogenemia in women with PCOS. There was a significant relationship between the circulating levels of free testosterone and the A allele of rs9939609, suggesting that the FTO mutation is vital for obesity and diabetes and for hyperandrogenism in PCOS women. Studies of European women with PCOS and Sri Lankan women confirm this [82, 96]. Subsequent experiments found that FTO expression was higher in PCOS and was positively related to androgen but negatively related to m6A. The dehydroepiandrosterone (DHEA) rat model demonstrated that FTO was overexpressed in the serum and ovarian tissues of hyperandrogenic PCOS. Cell experiments showed that dihydrotestosterone (DHT) could promote FTO expression, and FTO could influence the expression and activity of androgens. The coexistence of FTO and AR has been observed in vitro [97]. As a subsection of the steroid hormone receptor family, AR is abnormally expressed in PCOS patients with endometrial diseases [98], and increased AR coactivators, AR expression, and amplification in the endometrium of patients with PCOS have subsequently been demonstrated [99]. AR activation induces FTO upregulation, and the interaction of upregulated FTO promotes the expression of the AR signaling pathway and steroid synthase with AR, creating a positive feedback mechanism that promotes hyperandrogenemia in PCOS. FTO inhibitors were also found to prevent androgen excess and cell hyperproliferation by blocking the AR-AKT signaling axis in GC [98] (Fig. 2D). The association of the remaining m6A-related proteins with hyperandrogenemia in PCOS needs to be further investigated.

What’s more, the link between obesity, insulin and androgens requires further investigation. In PCOS, the sensitivity of adipose tissue to insulin has decreased, while that of androgen pathway has increased. Also obesity exacerbates the hyperandrogenic state of PCOS. abdominal obesity changes the clearance and deposition of fat-soluble androgen and increases hyperandrogenism by lowering sex hormone-binding globulin (SHBG) levels [100]. High insulin levels decrease hepatic SHBG levels and exacerbate the effect of LH on theca cell androgen production, increasing the blood concentrations of free and total testosterone [101]. Insulin has also been shown to stimulate testosterone production in adipose tissue through aldoketoredutase type 3 (AKR1C3) elevation and insulin resistance in women [102]. Addressing the linkages between the three, metformin can treat hyperandrogenemia by lowering insulin levels, whereas rosiglitazone may work by directly improving androgen sensitivity to insulin.

#### m6A-related proteins and anxiety and depression in PCOS

In the same way that depression is found to co-exist with chronic diseases, it seems that health professionals and women need to acknowledge the anxiety and depression in PCOS. Studies suggest that women with PCOS have a higher incidence of psychological disorders, including anxiety, depression, low self-esteem, and negative body image [6, 103]. An earlier study of 48 women aged 18–60 with PCOS who were administered the Anxiety and Depression Questionnaire showed that 34% of them reported having had a medical diagnosis of depression, and 21% had a medical diagnosis of anxiety. Moreover, 23% of them were taking prescription drugs for either anxiety or depression [6]. At the same time, their QOL scores tended to drop, with concerns about weight and infertility having the greatest negative impact, while hyperandrogenism, obesity, and infertility are weakly correlated with anxiety and depression [104]. In addition, a previous community-based study found that PCOS was also associated with a high prevalence of eating disorders and a high prevalence of bulimia nervosa compared to a control group [105].

It has been found that FTO-deficient mice have reduced anxiety and depression symptoms and that their anxiety and depression desensitisation symptoms are strongly associated with gut microbes. Certain gut microbes have been reported to maintain neurological homeostasis through the immune system [106, 107]. The study also revealed that lactobacilli play an important role in reducing anxiety-depression-like behaviors in mice and that anxiety-depression desensitization symptoms in FTO-deficient mice are closely related to gut microbes [108]. However, there is limited research on the direct link between m6A and psychiatric symptoms of PCOS, and most of them are caused indirectly by m6A-mediated obesity or hyperinsulinemia or by patients' anxiety about changes in body image. Therefore, further exploration is required.

#### m6A-related proteins and gut flora in PCOS

It is well known that the intestinal flora is the "endocrine organ" that maintains the health of the human body and maintains the homeostasis of the reproductive endocrine system by interacting with sex hormones, insulin, and so on [109]. Gut flora also plays a key role in regulating energy balance and is involved in the development of obesity and metabolic syndrome [110, 111]. Microbial metabolites can reach any part of our body through the somatic circulation after absorption. As a result, disruption of the gut microbiota network often affects multiple systems in the body, leading to a variety of diseases such as neurological disorders, inflammatory bowel disease and metabolic disorders. In recent years, gut flora has been shown to be directly related to or play a role in a variety of diseases, such as obesity and diabetes [112]. Some researchers have proposed the Dysbiosis of the Gut Microbiota (DOGMA) theory, suggesting that gut microbes may contribute to the typical features of PCOS [113]. In patients with PCOS, gut flora is associated with the development of chronic inflammation, insulin resistance, metabolic syndrome, and hyperandrogenism, and may alter the clinical features of PCOS through short-chain fatty acids, bile acids, androgens, and the cerebral-gut axis [114, 115].

Experiments have further demonstrated that the intestinal microbiome could be involved in the mechanism of PCOS through m6A [30]. It has been hypothesised that the intestinal flora may alter the stability of the intestinal mucosa by participating in the development of the inflammatory response in patients with PCOS, which in turn affects its metabolism. Recently Yang et al. demonstrated with different antibiotic treatments that intestinal flora can indeed interfere with remodeling the mouse liver, cecum and brain mRNA m6A epitope transcriptome in a tissue-specific manner Among them, writer METTL3 and METTL14 were most significantly downregulated, while reader YTHDC1 and eraser FTO were also downregulated in most of the antibiotic-induced gut ecological dysregulation. In contrast, under normal conditions, bile acids of microbial origin can maintain mRNA methylation levels and gene expression by regulating m6A levels after uptake into the circulation [116]. It has also been found that gut flora coordinates PCOS through the gut microbiota-bile acid-interleukin 22 axis, and that gut flora is involved in the regulation of bile acid-mediated IL-22 production through the GATA3 signaling pathway, which in turn affects ovarian function and insulin sensitivity in PCOS patients [117, 118] (Fig. 2A). The effect of intestinal flora on other m6A-related proteins remains to be investigated.

### Comprehensive treatment for specific symptoms of PCOS

#### Menstrual cycle abnormalities and ovulation disorders

Menstrual cycle abnormalities like oligo-ovulation, anovulation, and decreased progesterone exposure increase the risk of endometrial hyperplasia and malignancy [119]. On one hand, for women with no short-term need for reproduction, follow-up is sufficient for mild symptoms, while when menstruation occurs less than four times per year, a combination of oral contraceptives is recommended to regulate the menstrual cycle, especially if contraception is necessary or treatment for hyperandrogenism is required. The World Health Organization's guidelines are applicable, including the use of low-dose, naturally occurring lecithin and drugs with a low thromboembolic risk [120]. The main anti-androgenic effect of oral contraceptives is an increase in SHBG and a decrease in blood androgens, not a direct anti-androgenic effect of certain progestins in these preparations. Besides, as a biguanide used to treat T2DM, metformin has been widely used to treat PCOS. In terms of weight loss, fasting glucose and fasting insulin, combination metformin showed more significant improvements than either treatment alone [121]. In a randomized controlled trial of metformin and placebo, metformin significantly improved BMI, HOMA-IR and fasting glucose, and improved waist-to-hip ratio and lipid profile [122]. Although metformin improves ovulation disorders, insulin resistance, cycle control, and metabolism, it does not affect hyperandrogenism [123, 124]. On the other hand, for women of childbearing potential, immediate referral for assisted reproduction such as in vitro fertilization should be made if the partner has fertility problems. Ovulation promotion is recommended when ovulation is unpredictable or absent [125]. Metformin was also found to have a positive impact on pregnancy outcomes in women with PCOS. A study conducted a meta-analysis of 1606 pregnant women with PCOS and found that women who were treated with metformin throughout their pregnancy had a higher probability of having a full-term delivery, a vaginal delivery, and a lower probability of having a miscarriage, hypertensive syndrome during pregnancy, and gestational diabetes mellitus [68]. Similar to metformin, melatonin has shown beneficial effects in improving ovarian and uterine morphology and histology, but further clinical studies are needed to confirm these findings [126].

Moreover, adherence to a balanced diet and the intake of essential nutrients can counteract the risk of ovulation disorders. Diet-related factors are vital for regulating ovulation [127–129]. Dietary components can regulate ovulation through various pathways, like metabolic pathways, endocrine profiles, and carbohydrate metabolism. Adequate intake of low-glycemic index carbohydrates, adequate plant proteins, and unsaturated fatty acids is important. Adequate amounts of vitamins and minerals are necessary. In contrast, high glycemic index carbohydrates and saturated fatty acids should be limited [130].

#### Hairiness, alopecia and acne

Androgenic alopecia is among the toughest manifestations of hyperandrogenism, and reversal of final hair loss on a medical basis is rare. The goal of treatment is to limit hair loss progression. The results of hair loss can be improved by hair styling, hair replacement and addition, hair transplants [131] or new techniques (growth factors from platelet-rich plasma [132, 133] or stem cell-based therapies [134]). Optimal treatment of hirsutism involves hair removal (electrolysis, laser, and shaving) and lowering or exposure to androgens. Low-dose ethinyl oestradiol combined oral contraceptive pills (COCPs) are the treatment of choice for hirsutism in adults and adolescents because they increase SHBG levels and decrease LH, serum testosterone, and androstenedione levels [135]. For facial hirsutism, topical application of the ornithine decarboxylase inhibitor—eflornithine can lower the rate of hair growth. It may be used alone (when hirsutism is not prevalent) or in combination with oral contraceptives [124]. If poor results are observed after at least six months of COCP and/or cosmetic treatment [5], anti-androgens (antiandrogenic drugs such as spironolactone, flutamide, finasteride) may be considered for the treatment of hirsutism in women with PCOS, as well as bariatric surgery. Acne can be treated with dermocosmetics [136], dermabrasion [137], laser or light therapy [138], or cosmetic surgery for severe scarring. Among patients with PCOS, acne is often serious and persistent, demanding additional therapy with a combination of oral contraceptives [124, 139].

#### Depression and anxiety

As depression, anxiety and eating disorders can affect a patient's ability to engage in effective lifestyle management, there is a need to screen for these disorders to make individualized lifestyle recommendations [5]. For psychological symptoms like anxiety or depression, psychological profile screening is the first step in identifying and treating patients. In most cases, there is a heightened level of mental distress, clinical anxiety, and depression symptoms, which are largely affected by the multiplicity of PCOS characteristics. When first-line treatments for PCOS, such as COCP or lifestyle management, show slight or no improvement in anxiety-depression, this suggests that traditional approaches should be used when necessary, including psychotherapy and antidepressants or anxiolytics [140, 141]. Recognizing psychological characteristics and raising patients' psychological resilience by providing them with more knowledge of their state of health and their treatment may help to reduce mental illness. However, if the symptoms are serious, it is necessary to search for psychological or psychiatric treatment [17]. Therefore, the combination of cognitive behavioral therapy and lifestyle management may be an effective strategy to settle weight management in patients with PCOS and high depression scores [141, 142].

#### Regulating intestinal flora

Chinese herbs are commonly used in the treatment of PCOS. Modified Banxia Xiexin Decoction can regulate the disturbance of the diversity of intestinal flora [143]. Berberine can effectively improve the pathology of PCOS by regulating the intestinal microbiota and metabolites [144]. Guizhi Fuling Wan (GFW) can improve insulin resistance in PCOS by controlling inflammation through regulating intestinal flora [145]. In addition, GFW could inhibit granulosa cell autophagy and promote follicular development, thereby alleviating ovulation disorder in PCOS-IR rats, which was associated with the activation of PI3K/AKT/mTOR signaling pathway [146]. Oral quercetin appears to regulate LH hormones by decreasing inflammatory factors and oxidative stress, benefiting the treatment of PCOS [147]. Similarly, tempol improves the PCOS phenotype by reducing intestinal oxidative stress, restoring gut flora dysbiosis, and modulating interactions between the gut microbiota and host metabolites [148]. Probiotics, prebiotics and synbiotics are also among the effective treatment options for PCOS patients [149]. In addition, metformin, thiazolidinediones, and statins can also be used to treat PCOS by improving the characteristics of the gut flora [150]. Of course, since gut flora is strongly geographically specific, localities can explore the flora and standards that work for them. For milder cases it may be possible to start with dietary therapy to improve the gut flora, while more severe cases try medication and combination therapy.

#### Translational

As mentioned above, although oral contraceptives and progesterone have been used as primary therapeutic agents, their effects and populations are limited, and monitoring of blood glucose and lipids is required while taking them [131]. Studies have found that treating patients with pemphigus vulgaris with platelet-rich plasma can yield significant results, but more clinical evidence is needed [132]. Similarly, growth factor-rich plasma has been found to dermal papilla cell mitogenesis through regulating kinases/Akt pathway activation and the cell cycle– involved CDK4/cyclin D1 overexpression. And it has also been found to promote the renewal of connective tissue in the scalp. However, further research is needed to elucidate the applicability of its treatment modalities [133]. Another study showed that injections of human follicle stem cells preparations had a significant therapeutic effect on male androgenetic alopecia, but further clinical practice on a larger scale is needed [134, 151].

#### Targeted therapy against m6A

**Fig. 3:**
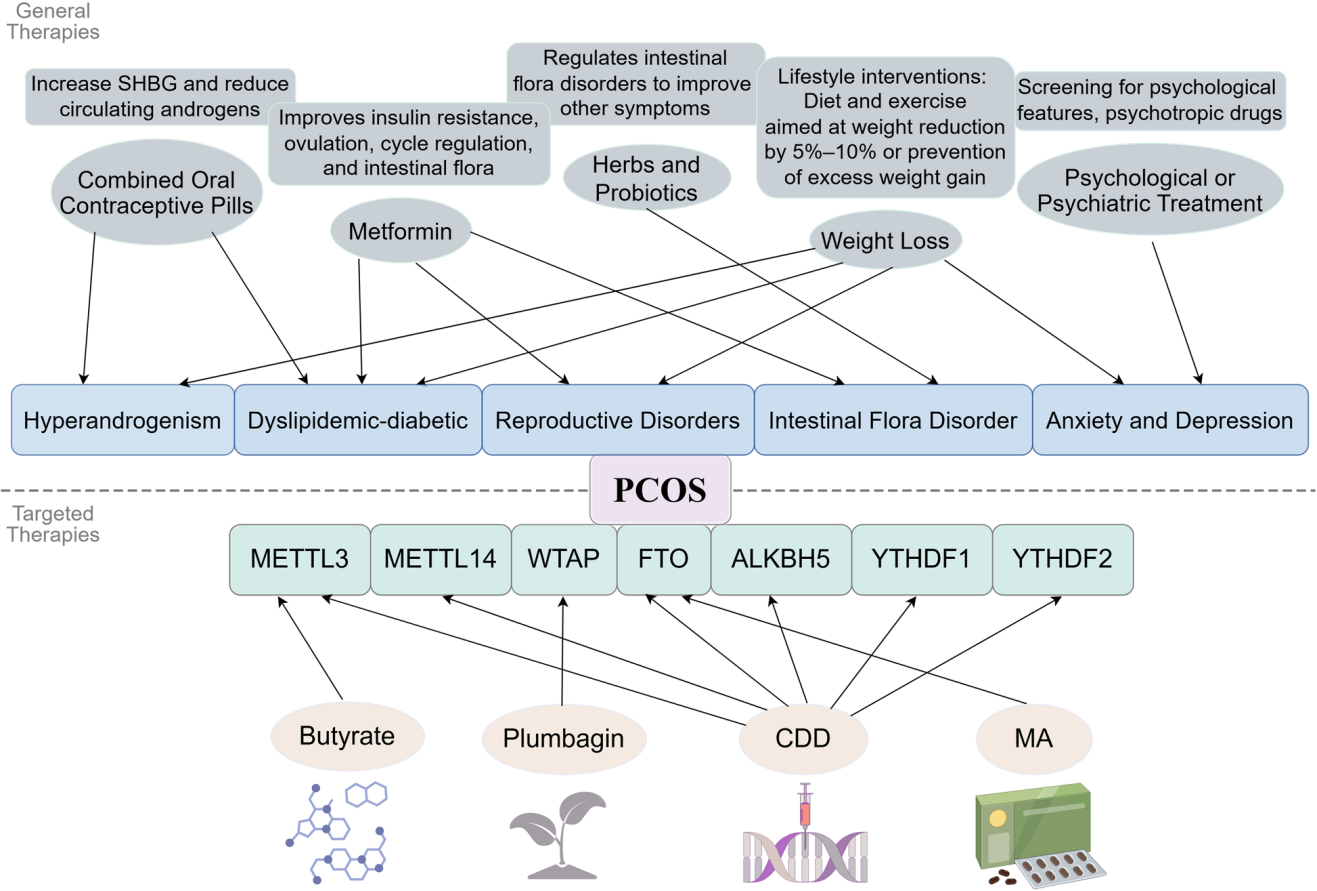
General and targeted m6A therapies for PCOS. Abbr: CDD, CangFu DaoTan Decoction; MA, FTO-specific inhibitor; ALKBH5, alkB homolog 5; YTHDF, YTH domain family proteins

Butyric acid is the most abundant short-chain fatty acid and the main source of energy for colon cells, in addition to its anti-inflammatory, antioxidant and anti-cancer properties [152]. It was found that the levels of acetic, propionic, and butyric acids were significantly lower in PCOS patients than in the control group, and butyric acid decreased more in obese PCOS patients compared to the other two groups [153]. As a product of the digestive process of intestinal microorganisms, butyric acid promotes the secretion of gut hormones by interacting with receptors on the surface of intestinal epithelial cells [154]. Previous study has noted that the microbiome strongly influences the expression of host m6A mRNA modifications in the brain, and the authors also detected strong changes in the expression of the methyltransferase METTL3 in the brain [155]. An experiment later found that butyric acid was reduced in women with PCOS and was regarded as a potential METTL3 target. The researchers divided the PCOS mouse model into four groups—control, obese PCOS, low-dose butyric acid and high-dose butyric acid and found that the high-dose butyric acid group had a significantly lower number of follicles of different sizes in the ovaries compared to the PCOS group. By inhibiting METTL3 expression, butyric acid ameliorated LPS-induced apoptosis and oxidative stress and suppressed the expression of inflammatory cytokines, for example, PPAR-γ and GLUT4 levels were reduced in the PCOS model mice, but administration of butyric acid resulted in elevated levels of PPAR-γ and GLUT4 as well as reduced levels of IL-6, NLRP3 and TNF-α [30] (Fig. 3). Short-chain fatty butyric acid is expected to be an effective therapy for PCOS.

Jing et al. showed that FTO inhibitor MA combined with AKT inhibitor LY294002 can inhibit GC cell survival by inducing cell cycle arrest. FTO is highly expressed in human brain tissue and glandular tissues such as thyroid, endometrial and ovarian tissues and has been shown to be associated with cardiovascular disease, type 2 diabetes mellitus and other metabolic disorders [78, 156, 157]. In addition, it is also involved in the regulation of ovarian function through m6A demethylation in the process of ovarian aging [158, 159]. Moreover, genetic variants of FTO are associated with insulin resistance characteristics in PCOS patients [83, 160]. Therefore, inhibition of FTO might be effective in improving the symptoms of PCOS, and they finally demonstrated that the combination of MA and LY294002 inhibited the growth of GCs characterized by hyperandrogenaemia in PCOS. The combination of inhibitors MA and LY294002 may be a new PCOS treatment modality [81] (Fig. 1D).

Green cardamom contains various beneficial components like flavonoids (quercetin and camperfor), flavonoids (lutolin), and anthocyanins and has antioxidant and anti-inflammatory properties [161]. By inhibiting oxidative stress, green cardamom may influence insulin sensitivity, inflammation, and hepatic stasis [130]. A double-blind randomized controlled trial (RCT), the first to assess the impact of green cardamom medication on the expression of obesity and diabetes genes in women with PCOS, showed that green cardamom intervention improved glucose indices, anthropometric indices, and sex hormones, and FTO, leptin receptor (LEPR), carnitine palmitoyltransferase 1 (CPT1A), and PPAR-γ genes in women with PCOS. While in animal models, CPT1A was associated with elevated WC, BMI, and hypertriglyceridemia [162, 163]. LEPR polymorphism has been shown to be associated with insulin resistance, obesity, dyslipidemia, and increased serum leptin levels in women with PCOS due to high-fat content [164, 165].

A study by Cai et al. found that Plumbagin treatment was effective in reducing focal death in GCs. Plumbagin is the active compound of Plumbago zeylanica L. [31]. Over the last couple of decades, research has shown that plumbagin may be involved in various cancers [166, 167]. Plumbagin has been shown to inactivate the main pathways associated with the proliferation of cancer cells, including matrix metalloproteinase-9 (MMP-9), Akt/NF-κB, and vascular endothelial growth factor, thereby preventing cancer progression [167]. Plumbagin has also been shown to prevent PCOS development in experimental rats because it can reduce the pathological level of apoptosis in patients with PCOS [168]. The researchers treated PCOS mice with intraperitoneal injections of Plumbagin and found that during the development of PCOS, a large number of GCs pyroptosis as a result of hyperactivation of caspase-1 inflammasomes. Furthermore, they found that overexpression of the RNA N6-methylase compound WTAP in GC could stabilize ASC mRNA so that GC pyroptosis could be induced in PCOS. Taken together, these results suggest that plumbagin can inhibit WTAP from methylating ASC, thereby reducing GC pyroptosis and PCOS progression [32] (Fig. 3).

The traditional Chinese medicine formula CangFu DaoTan Decoction (CDD) has been reported to significantly reduce body weight, blood glucose levels, and LH and T levels in PCOS rats. CDD was found to reduce the expression of METTL3, METTL14 [151], FTO, ALKBH5 [169], YTHDF1 and YTHDF2 in peripheral blood and ovarian tissues of PCOS rats, as well as the expression of METTL3, FTO and YTHDF1 proteins in ovarian tissues [170] (Fig. 3). Similarly, studies have found spearmint oil to restore follicular maturation and induce ovulation through anti-androgenic, cholesterol-lowering and antioxidant properties [171]. In addition, many herbs phytochemicals have shown potential or proven efficacy in treating PCOS in preclinical studies, and more research is needed to determine their dosage and efficiency [172].

## Conclusions

The role of m6A modification is becoming clear with the development of high-throughput sequencing technologies and highly specific antibodies against m6A. In this review, we summarized the processes and mechanisms that m6A-related proteins involved in ovulatory disorders, abnormalities of glucose and lipid metabolism, and hyperandrogenemia, respectively, mainly from the perspective of the clinical features of PCOS. Furthermore, Butyric acid, Plumbagin, and MA as m6A-based targeted therapies for PCOS were illustrated at the molecular level. Secondly, it has been found that one m6A protein may contribute to several clinical phenotypes, such as FTO, and several m6A proteins may contribute to one phenotype. We hypothesize that m6A-related proteins may be used to categorize the clinical phenotypes of several female reproductive disorders and to predict the interconnections between and inter-conversion of these phenotypes. Nowadays, there are more and more studies on m6A in PCOS, but except for the major m6A proteins METTL3, FTO, YTH family, the rest of the regulatory factors, such as METTL14 and ALKBH5, are less studied and not much clinically translated, so there is still an emergent need to explore the role of other m6A-related proteins or investigate the long-term effects of m6A-targeted therapies. We hope that this article can provide some references for subsequent researchers.

## Data Availability

No datasets were generated or analysed during the current study.

## Abbreviations

PCOS: Polycystic ovary syndrome
m6A: N6-methyladenosine
METTL3/14: Methyltransferase-like 3/14
VIRMA/KIAA1429: Vir-like m6A methyltransferase-associated
WTAP: Wilms-tumor associating protein
RBM15 A/B: RNA-binding motif protein 15
ZC3H13: Zinc finger CCCH domain-containing protein 13
CBLL1/HAKAI: Cbl proto-oncogene E3 ubiquitin protein ligase-like 1
FTO: Obesity-associated protein
ALKBH5: AlkB homolog 5
YTHDF1/2/3: YTH domain family proteins
YTHDC1/2: YTH domain-containing proteins 1–2
HNRNPA2B1: Heterogeneous nuclear ribonucleo-protein A2B1
IGFBP1/2/3: Insulin-like growth factor 2 mRNA binding proteins
eIF3: Eukaryotic initiation factor 3
LRPPRC: Leucine Rich Pentatricopeptide Repeat Containing
EHT: Endothelial-to-hematopoietic transformation
HSPCs: Hematopoietic stem/progenitor cells
MII: Metaphase II
GVBD: Germinal vesicle breakdown
LH: Luteinizing hormone
AMH: Anti-Müllerian hormone
TNF-α: Tumor necrosis factor-α
TRAF6: Tumor necrosis factor receptor-associated factor
LPS: Lipopolysaccharides
TGF-β1: Transforming Growth Factor Beta-1
KGN cells: Human ovarian granulosa tumor cells
ASC: Caspase-recruitment domain
GC: Granulosa cells
AN: Arcuate nucleus
SNPs: Single-nucleotide polymorphisms
SHBG: Sex hormone-binding globulin
GLUT4: Glucose transporter type 4
GnRH: Gonadotropin-releasing hormone
AKR1C3: Aldoketoredutase type 3
AR: Androgen receptor
QOL: Quality-of-life
RCT: Randomized controlled trial
LEPR: Leptin receptor
CPT1A: Carnitine palmitoyltransferase 1
DEHA: Dehydroepiandrosterone
